# Pericardial Effusion in Erdheim-Chester Disease: A Case Report and a Mini Literature Review

**DOI:** 10.7759/cureus.22010

**Published:** 2022-02-08

**Authors:** Juhaina Al Hinai, Maitha Al Sibani, Juhaina S Al-Maqbali, Abdullah M Al Alawi

**Affiliations:** 1 Internal Medicine Training Program, Oman Medical Specialty Board, Muscat, OMN; 2 Clinical Pharmacy, Sultan Qaboos University Hospital, Muscat, OMN; 3 Medicine, Sultan Qaboos University Hospital, Muscat, OMN

**Keywords:** retroperitoneal fibrosis, pericardial tamponde, pericardial effusion, histiocytosis, erdheim-chester disease

## Abstract

A 68-year-old man diagnosed with Erdheim-Chester disease presented to the emergency department with shortness of breath of one-day duration. Upon presentation, the patient was dyspnoeic and hypoxemic. The initial laboratory workup showed raised inflammation markers, and a chest x-ray showed the presence of bilateral lung infiltrates; therefore, he was managed for community-acquired pneumonia with antimicrobial and other supportive measures. Due to lack of improvement, he had transthoracic echocardiography (ECHO), which showed a large pericardial effusion without tamponade. He was treated with corticosteroids and underwent pericardiocentesis, which resulted in remarkable symptomatic improvement. This case presents a serious manifestation of a rare disease and summarizes treatment options from the literature.

## Introduction

Erdheim-Chester disease (ECD) is a rare disease first described in 1930 and characterized by the presence of polymorphic granuloma infiltrated by foamy histiocyte and fibrosis [1, 2]. ECD manifestations include painful long bone osteosclerosis with endocrinopathy, interstitial lung disease, cutaneous manifestation neurodegenerative manifestations, and retroperitoneal fibrosis [2-4]. Also, ECD has serious cardiovascular manifestation coated aorta, coronary stenosis, myocardial infarction, pericarditis, and pericardial effusion with or without cardiac tamponade [2-4]. ECD's cardiovascular manifestations are common and associated with poor prognosis [2, 5]. This case reports about a patient who presented with a large pericardial effusion. It also reviews the therapeutic approach based on the previously reported cases in the medical literature.

## Case presentation

A 68-year-old man presented to the emergency department at Sultan Qaboos University Hospital (SQUH) with a one-day history of worsening shortness of breath. He denied any fever, cough, or chest pain. His medical background included hypertension, chronic kidney disease, and ischemic heart disease that required percutaneous transluminal coronary angioplasty in 2009. In addition, he was diagnosed with ECD in 2018, after he presented with orthostatic hypotension due to newly introduced antihypertensives. During that admission, he was found to have lower lobe consolidation and nodules in the chest x-ray, which triggered a series of investigations, that concluded a diagnosis of ECD with negative v-raf murine sarcoma viral oncogene homolog B1 (BRAF) mutation (Table 1).

**Table 1:** Diagnostic workup leading to the diagnosis of Erdheim-Chester disease in 2018 FDG: fluorodeoxyglucose, Pan-CK: pan Cytokeratin antibodies, CD1a: Cluster of Differentiation1a, BRAF: v-raf murine sarcoma viral oncogene homolog B1, NRAS: neuroblastoma rat sarcoma virus,

investigation	Results
High resolution computed tomography of the chest	Multiple small lung nodules (peri-lymphatic and sub-pleural) Atelectatic lung lesions Mild bilateral pleural effusion Small pericardial effusion
Computed tomography of abdomen and pelvis	Multiple small mesenteric and para-aortic lymph nodes The left kidney is small with a simple cyst Right kidney normal in size, with few cysts
Magnetic resonance imaging of the brain	Few intracranial intra-axial lesions
Positron emission tomography	FDG avid bilateral small lung nodules and nodular septal thickening with FDG avid atelectatic lung lesions FDG avid widespread bone lesions FDG avid brainstem lesion Focally increased FDG activity in the aortic wall at several levels
Biopsy of lung nodules and left acetabular lytic lesion	A fibroinflammatory process, with infiltration of histocytes, fibroblasts, foamy histocytes, and giant cells. Immunohistochemistry: Histocytes are positive for Vimentin and CD68 and are negative for Pan-CK, and CD1a Ki67 is very low
Genetic mutations	BRAF^V600E ^and NRAS are not detected
Echocardiography	Ejection fraction 50-70% with good contractility. No valvular abnormality. Minimal posterior and lateral pericardial effusion measuring 0.6cm, without tamponade effect.

ECD management included six weekly of injection infliximab, oral azathioprine 50 mg (two times per day), and prednisolone (5 mg daily), and his condition remained stable until his recent presentation to the emergency department. 

Upon presentation, his vitals were as follows: temperature 37.9 centigrade, heart rate 90 beats per minute, respiratory rate 32 breaths per minute, blood pressure 193/100 mm Hg, oxygen saturation 87% on ambient air. On examination, he was in distress, with significant crepitation all over his chest but no pedal edema. His inflammatory markers were high, and chest x-ray showed bilateral reticulonodular opacities, increased vascular markings, and bilateral pleural effusion. COVID-19 RNA was not detected, and a workup for atypical pneumonia was done (Table 2).

**Table 2:** Laboratory tests results upon presentation to the hospital GFR: glomerular filtration rate

Haemoglobin (g/dL)	10.4	11.5-15.5
White blood cells count (x10^9^/L)	11.7	2.2-9.0
Platelet (x10^9^/L)	480	150-450
C-reactive protein (mg/L)	112	0-10
Serial troponin (ng/L)	88 – 57 – 45	<14
Sodium (mmol/L)	136	135-145
Potassium (mmol/L)	4.9	3.5-5.1
Bicarbonate (mmol/L)	19	21.8-26.9
Chloride (mmol/L)	100	98-107
Magnesium (mmol/L)	0.72	0.66-0.89
Corrected calcium (mmol/L)	2.42	2.15-2.55
Phosphate (mmol/L)	1.24	0.81-1.45
Creatinine (umol/L)	188	59-104
Estimated GFR (mL/min/1.73m^2^)	31	>90
Urea (mmol/L)	7.9	2.8-8.1
Anion gap (mmol/L)	17	5-13

**Figure 1:**
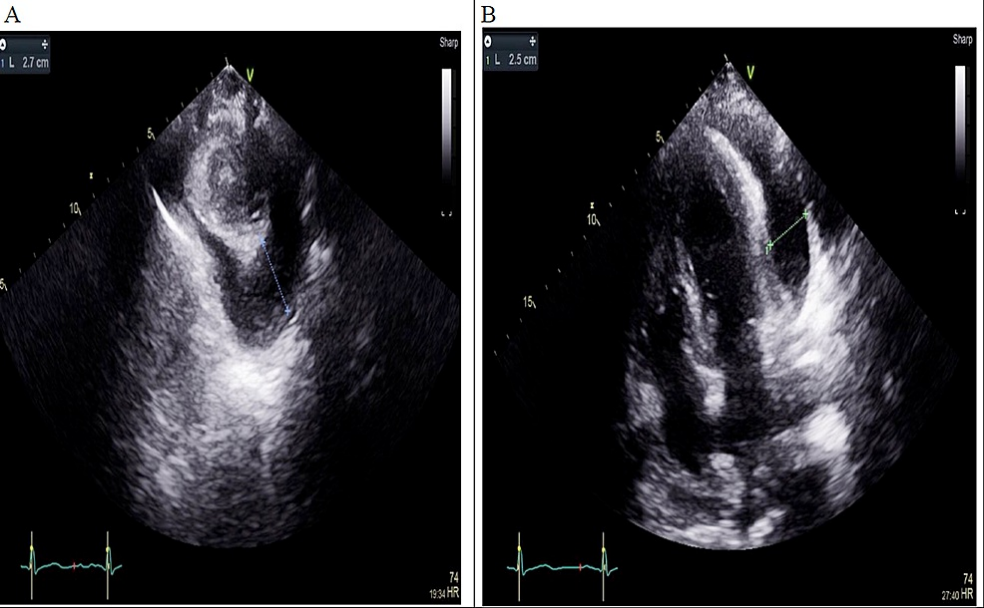
Parasternal short axis window (A) and apical 4 chamber window show large pericardial effusion (B).

He was treated with antimicrobials (IV ceftriaxone, oral azithromycin, and oseltamivir) and an O2 supplement. He had transthoracic echocardiography, which demonstrated a large pericardial effusion measuring 2.7 cm posteriorly, without tamponade effect (Figure 1).

He was treated with 1 gram of methylprednisolone for three days, followed by ultrasound-guided percutaneous pericardiocentesis due to ongoing symptoms. A round 500 ml of straw-colored pericardial fluid was drained and analysis showed leukocytosis and erythrocytes with no bacterial growth from culture. In addition, cytology revealed high mesothelial cells and macrophages that could not be distinguished from normal macrophages (non-Langerhans cells).

After the pericardiocentesis, the patient had symptomatic improvement accompanied by improvement in his oxygen requirement during the hospital stay. Therefore, he was discharged with 50 mg of prednisolone (to be tapered by 5 mg every week), and he was booked to be reviewed in an outpatient clinic.

## Discussion

ECD is an extremely rare disease of non-Langerhans cell histiocytosis; it was first described by William Chester and Jakob Erdheim and characterized by uncontrolled chronic inflammation [2, 6, 7]. The oncogenic BRAF (V600E) mutation was reported in 54% of the cases [5, 6, 8]. The disease progress through fibro-inflammatory infiltrates to tissue organ by the presence of foamy histiocytes, multinucleated giant cells, and lymphocytes [6, 9]. The manifestations of ECD in this patient at the time of diagnosis included the presence of lesions in the brain stem, lung, bones, para-aortic lung nodules, and evidence of minimal pericardial effusion. Overall, cardiac involvements are present in 75% of patients with ECD, and it is associated with worsening disease prognosis leading to death in around 60% of the patients. The most common cardiac involvement is pericardial effusion, leading to tamponade [10]. The only treatment option approved by FDA in 2017 for ECD patients with positive BRAF (V600E) mutation is BRAF kinase inhibitor (oral vemurafenib) [11, 12].

On the other hand, a literature review revealed various immunosuppressive treatments used to treat patients with negative mutation BRAF (V600E). These treatments included interferon alfa, steroids, methotrexate, infliximab, or oral azathioprine as monotherapy or in combination, as described by several case reports [8, 13]. ECD prognosis is affected by the type of organ involved. For example, central nervous system defects, which occur mainly in older patients and cardiovascular lesions, are associated with poor prognosis, yet the prognosis varies provided that patients get supportive care as early as possible [14]. To date, there are 31 case reports and four case series published in the literature describing the management of pericardial effusion in patients with ECD. Percutaneous pericardiocentesis is the most common intervention used to remove pericardial effusion, while the pericardial window is used only in case of recurrence [4, 7, 15]. Nevertheless, interferon alfa is considered the first-line option for management of ECD, for its associated improvement in patients’ five-year survival (82.7%) [13, 14].

## Conclusions

ECD is a non-Langerhans is a rare cell histiocytosis that involves fibro-inflammatory effects associated with multi-organ involvements. Pericardial effusion is a severe manifestation of ECD. We described a successful therapeutic approach that included systematic steroids and pericardiocentesis to treat a large pericardial effusion.
